# Psychological Results of 438 Patients with persisting Gastroesophageal Reflux Disease Symptoms by Symptom Checklist 90-Revised Questionnaire

**DOI:** 10.5005/jp-journals-10018-1230

**Published:** 2017-09-29

**Authors:** Xia Chen, Ping Li, Fei Wang, Guozhong Ji, Lin Miao, Sihong You

**Affiliations:** 1 Department of Gastroenterology, The Second Affiliated Hospital of Nanjing Medical University, Nanjing, Jiangsu, China

**Keywords:** Gastroesophageal reflux disease, High-resolution manometry, Impedance-pH monitoring, Refractory proton pump inhibitor symptoms, Symptom checklist 90-Revised questionnaire.

## Abstract

**Aims and objectives:**

Gastroesophageal reflux disease (GERD) affects mental state and social activities. On the contrary, mental disorders may also play a crucial role in GERD symptoms. The purpose of the study was to analyze the data of Symptom Checklist 90-Revised (SCL-90-R) questionnaire from patients with persisting GERD and to explore the impact of psychological factors on them.

**Materials and methods:**

The patients accepted SCL-90-R questionnaire survey, following endoscopy, high-resolution manometry (HRM), and ambulatory impedance-pH monitoring. Based on these results, we divided patients into different groups. The result of SCL-90-R was also compared with degree of acid reflux, symptoms, symptom duration, and gender.

**Results:**

The data from 438 patients were analyzed. All patients were divided into reflux esophagitis (RE; 63, 14.38%); nonerosive gastroesophageal reflux disease (NERD; 106, 24.20%); functional heartburn (FH; 123, 28.08%), hypersensitive esophagus (HE; 67, 15.29%), diffuse esophageal spasm (DES; 5: 1.14%), hypertensive (10, 3.42%); weak peristalsis (14, 3.20%); achalasia (50, 11.42%). There were significant differences between different groups regarding depression (DEP), anxiety (ANX), paranoid ideation (PAR), and psychoticism (PSY). The patients with ≥2 years with GERD presented with increased scores in DEP, ANX, and PSY. Women had dramatically higher scores than men in each domain (p < 0.05).

**Conclusion:**

Data have shown that GERD patients exhibit differential levels of psychological symptoms. Long duration of GERD was related to typical plus atypical symptoms and females seem to be more prone to develop psychological disorders.

**How to cite this article:** Chen X, Li P, Wang F, Ji G, Miao L, You S. Psychological Results of 438 Patients with persisting Gastroesophageal Reflux Disease Symptoms by Symptom Checklist 90-Revised Questionnaire. Euroasian J Hepato-Gastroenterol 2017;7(2):117-121.

## INTRODUCTION

Due to several factors, the incidence of GERD has been increasing in Asian and Western countries.^1,2^ Therapeutic strategy for GERD mainly involves the use of proton pump inhibitors (PPIs). Despite treatment with standard PPI dose, unfortunately, 20 to 30% of patients still suffer from troublesome GERD symptoms.^3^ Persisting GERD symptoms cause discomfort, impair quality of life, and affect mental state and social activities. Likewise, the subsequent development of mental disorders, such as ANX, and depressive symptoms also plays a crucial role on GERD symptom deterioration and has a negative effect on people’s quality of life.^4^

As a self-report questionnaire may be able to provide multidimensional and normative data, the SCL-90-R is widely used for all psychiatric patients. Some medical groups applied SCL-90-R to evaluate the psychological state of patients with chronic diseases.^5,6^ Previous studies suggest that the SCL-90-R could be valuable in evaluating psychological factors and, therefore, recommend it for psychosocial investigation in varied disease clinical practice.^7-9^ However, data from patients with persisting refractory GERD symptom based on SCL-90-R questionnaire have not been properly evaluated.

This study focused on the psychological state of refractory GERD patients using SCL-90-R. We intended to find the differences between several conditions of refractory GERD symptom and to assess if SCL-90-R would be a new, simple method for screening patients with GERD in a community hospital. Consequently, it may be helpful for gastroenterologists to choose relatively precise diagnostic approaches.

## MATERIALS AND METHODS

The target patients were all with persisting GERD symptoms after 8-week trial of once-daily PPI therapy from October 2010 to November 2015.

Exclusion criteria included previous psychopathological disease; previous digestive disease surgery; organic disease in digestive tract; severe comorbidity; any type of cancer; currently taking psychological drugs; and diagnosis of functional gastrointestinal disorders (FGIDs).

The tests, such as SCL-90-R,^10^ upper gastrointestinal endoscopy, and HRM (Given Imaging, Los Angeles, California) were accomplished; 24-hour impedance-pH monitoring (Given Imaging, Los Angeles, California) was done. Data of HRM and impedance-pH monitoring analysis were performed using software (Mano View software; Sierra Scientific Instrument Inc, Los Angeles, California).

The SCL-90-R consists of 90 items, evaluating psychopathological and somatic status on a 4-point scale. The scale ranges from 0 (absence of the symptom) to 4 (maximum disturbance). These items are divided into nine scale markers as: DEP; somatization (SOM); ANX; obsessive-compulsive behavior (O-C); hostility (HOS); interpersonal sensitivity (I-S); phobic anxiety (PHOB); PSY; and PAR. Every dimension includes 6 to 13 questions. The score on each dimension represents the mean score of the dimension and directly reflects the severity of the mental health problem. Subscale scores >2 were suggestive of potential mental health issues. The global severity index (GSI) indicates the mean score of 90 items.^11^

Based on endoscopy, HRM, and impedance-pH monitoring, we divided patients into subgroups, such as NERD, RE, FH, HE, DES, hypertensive, weak peristalsis, and achalasia. The diagnosis was made and classified according to the Chicago classification and previously published criteria.^12,13^

## STATISTICAL ANALYSIS

Data analysis was performed independently by two investigators unaware of the status of individuals. The data were shown as mean ± standard deviation. Analysis of variance for continuous variables and Pearson’s chi-square test for categorical variables using Statistical Package for the Social Sciences version 13.0 was accomplished. A p-value <0.05 was significant and all p values were two-sided.

## RESULTS

### Clinical Characteristics

A total of 438 patients (137 male, 301 female, age 51.42 ± 17.30 years) were analyzed in this study (Table 1). Symptom duration of these patients was 4.02 ± 1.45 years. There were 271 (61.87%) patients with heartburn and 239 (54.57%) with regurgitation, 189 (43.15%) with retrosternal discomfort and pain (Table 1). All patients were divided into RE (63, 14.38%), NERD (106, 24.20%), FH (123, 28.08%), HE (67, 15.29%), DES (5, 1.14%), hypertensive (10, 3.42%), weak peristalsis (14, 3.20%), achalasia (50, 11.42%).

**Table 1:** **Table 1:** Demographics and clinical characteristics

*Characteristics (n = 438)*		*n*		*%*	
*Age (years)*		51.42 ± 17.30			
Range		19-83			
*Gender*					
Male		137		31.28	
Female		301		68.72	
BMI		23.42 ± 10.37			
RE		63		14.38	
NERD		106		24.20	
HE		123		28.08	
FH		67		15.29	
DES		5		1.14	
Hypertensive		10		3.42	
Weak peristalsis		14		3.20	
Achalasia		49		11.19	
Smoking		142		32.42	
Alcohol consumption		145		33.10	
*Drug history*					
Calcium ion antagonist		179		40.87	
Aspirin		153		34.93	
Hypnotic drug		51		11.64	
Past medical history		253			
*Symptom*					
Duration (years)		4.02±1.45			
Heartburn		271		61.87	
Regurgitation		239		54.57	
Retrosternal discomfort and pain		189		43.15	
Cough		74		16.89	
Asthma		19		4.34	
Hoarseness		34		7.76	
Throat discomfort		195		44.52	
Foreign body sensation in throat		113		25.80	
Globus sensation		63		14.38	
Belching		204		46.58	
Dysphagia		152		34.70	
Epigastric pain and epigastric discomfort		204		46.58	

BMI: Body Mass Index

**Table 2:** **Table 2:** Scores in different groups

		*RE (n = 63)*		*HE (n = 67)*		*FH (n = 123)*		*NERD (n = 106)*		*Achalasia (n = 50)*		*Hypertensive (n = 10)*		*DES (n = 5)*		*Weak (n = 14)*	
SOM		1.39 ± 0.58		1.46 ± 0.62		1.57 ± 0.67		1.40 ± 0.62		1.54 ± 0.66		1.66 ± 0.72		1.20 ± 0.59		1.46 ± 0.90	
O-C		1.50 ± 0.54		1.42 ± 0.64		1.55 ± 0.63		1.50 ± 0.63		1.62 ± 0.73		1.39 ± 0.56		1.94 ± 0.70		1.62 ± 0.73	
I-S		1.43 ± 0.67		1.35 ± 0.66		1.38 ± 0.57		1.40 ± 0.59		1.48 ± 0.71		1.57 ± 0.75		1.20 ± 0.59		1.60 ± 0.69	
DEP*		1.48 ± 0.64		1.51 ± 0.65		1.77 ± 0.59		1.80 ± 0.59		1.76 ± 0.66		1.27 ± 0.54		1.65 ± 0.67		1.61 ± 0.81	
ANX*		1.50 ± 0.60		1.46 ± 0.70		1.69 ± 0.62		1.70 ± 0.53		1.87 ± 0.66		1.34 ± 0.57		1.57 ± 1.06		1.72 ± 0.73	
HOS		1.44 ± 0.56		1.40 ± 0.62		1.40 ± 0.61		1.39 ± 0.67		1.35 ± 0.56		1.40 ± 0.84		1.20 ± 0.59		1.51 ± 0.75	
PHOB		1.48 ± 0.64		1.47 ± 0.62		1.42 ± 0.58		1.47 ± 0.64		1.56 ± 0.68		1.20 ± 0.57		1.57 ± 1.06		1.37 ± 0.56	
PAR*		1.27 ± 0.49		1.16 ± 0.47		1.27 ± 0.47		1.32 ± 0.57		1.12 ± 0.44		0.96 ± 0.36		0.56 ± 0.51		1.36 ± 0.51	
PSY*		1.26 ± 0.47		1.31 ± 0.53		1.23 ± 0.51		1.27 ± 0.50		1.59 ± 0.57		1.58 ± 0.60		1.67 ± 0.48		1.51 ± 0.50	
GSI*		1.04 ± 0.41		1.01 ± 0.42		1.02 ± 0.38		1.18 ± 0.38		1.07 ± 0.44		1.17 ± 0.47		0.94 ± 0.43		1.11 ± 0.44	

*p < 0.05 between groups

### SCL-90-R Questionnaire Survey

We investigated the scores of SCL-90-R in different groups (Table 2). The results indicated that the scores had significant differences between various groups as shown by DEP, ANX, PAR, PSY, and GSI. In DEP domain, the score in RE group was less than that in NERD, FH, achalasia groups (p < 0.05); the score in NERD was more than in HE and hypertensive groups (p < 0.05); the score in FH was more than in hypertensive and HE (p < 0.05); the score in achalasia was more than in HE as well as hypertensive groups (p < 0.05). About ANX domain, we found RE group had less score than in NERD and achalasia groups (p < 0.05); NERD group had more than HE group (p < 0.05); FH group had more score than HE group (p < 0.05); achalasia had more score than HE and hypertensive groups (p < 0.05). For PSY, the score of achalasia group showed higher level than RE, NERD, FH, and HE groups (p < 0.05); the score of FH group was more than hypertensive group (p < 0.05). For PAR, NERD group had more score than HE, hypertensive and achalasia groups (p < 0.05), and hypertensive group had more score than DES group (p < 0.05). According to the result of GSI domain, the data revealed that the level of NERD was higher than RE, FH, and HE groups (p < 0.05).

**Graph 1:**
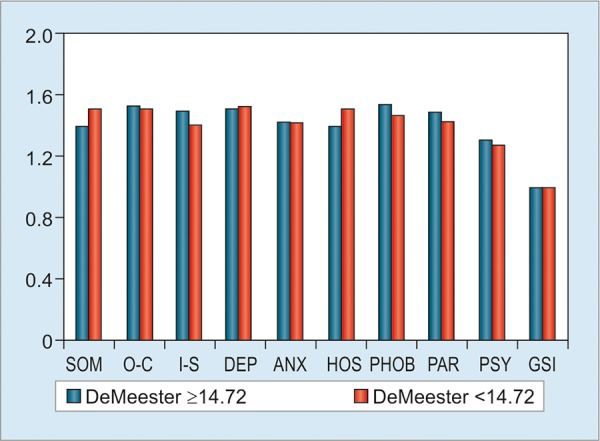
SCL-90-R scores based on DeMeester value. No differences were detected in nine domains and GSI between DeMeester value 214.72 and DeMeester value <14.72

**Graph 2:**
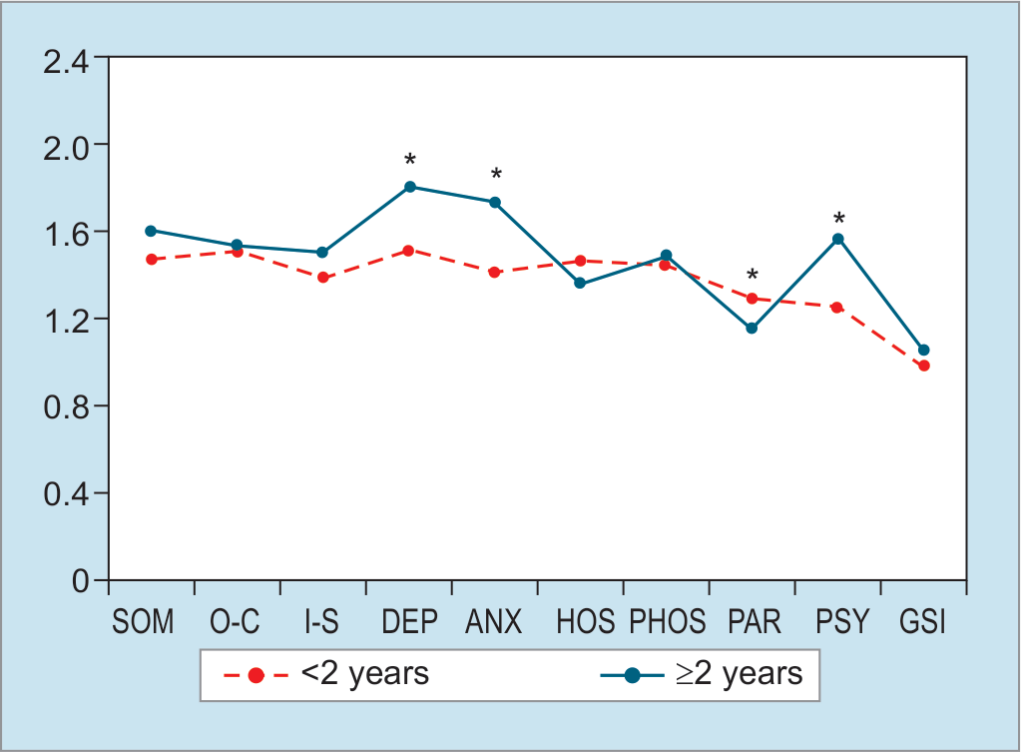
SCL-90-R scores depending on symptom duration. Significant differences were seen in DEP, ANX, PAR, and PSY domains. *p < 0.05

**Graph 3:**
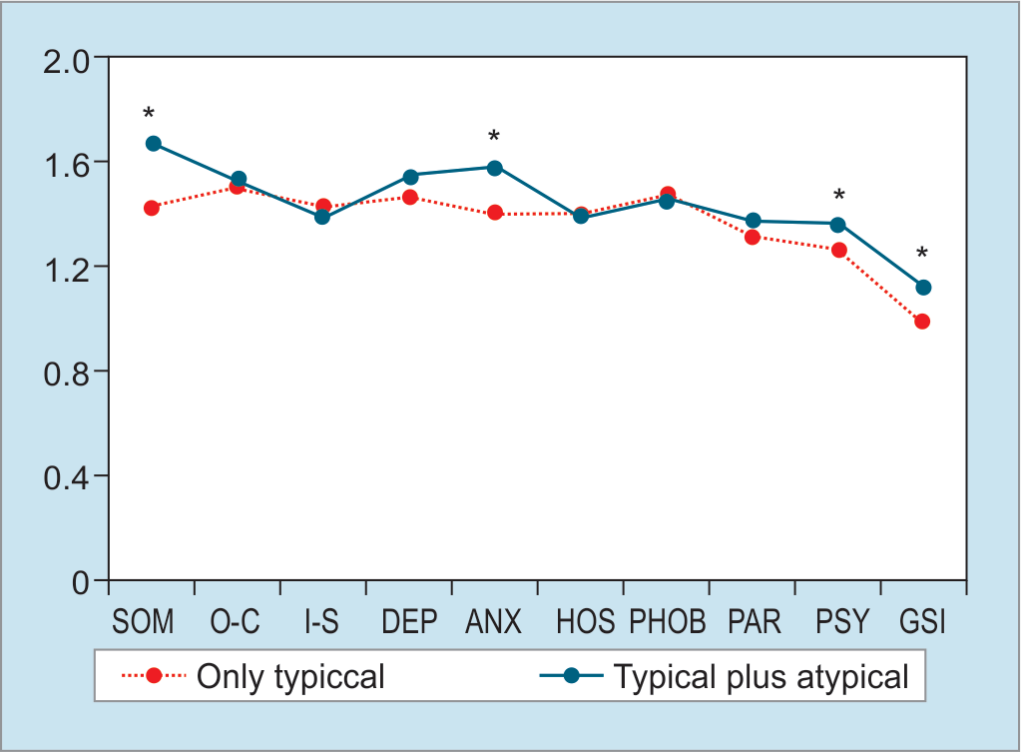
SCL-90-R scores based on symptom. Significant differences were seen in SOM, ANX, and PSY domains and GSI. *p < 0.05

**Graph 4:**
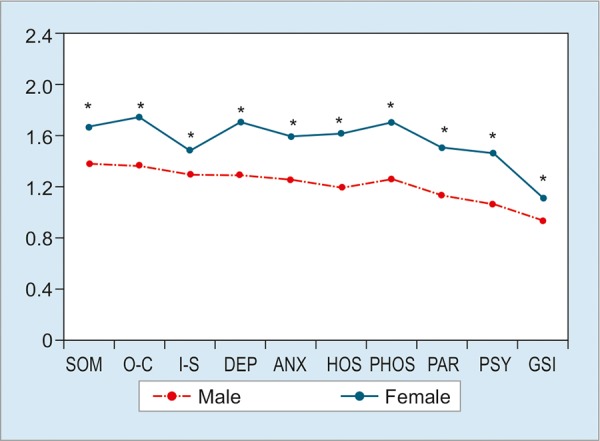
SCL-90-R scores based on gender. The scores of females were significantly more than those of males in all domains and GSI. *p < 0.05

The nine domains with DeMeester pathological (>14.72, n = 114) and normal (<14.72, n = 245) values showed no statistical differences (p > 0.05; Graph 1). However, we found statistically significant differences between the patients with less duration of GERD symptoms (<2 years, n = 95) with those with prolonged GERD duration (>2 years, n = 343). The patients with more duration presented more scores in DEP, ANX, and PSY (p <0.05; Graph 2). But the score of PAR was lower in >2 years group (p < 0.05). Subjects with GERD typical plus atypical symptoms (n = 253) had higher scores compared with subjects only with typical symptoms (n = 185) in SOM, ANX, PSY, and GSI (p < 0.05; Graph 3). Compared with gender, women had dramatically higher scores than men in these nine domains (all p < 0.05; Graph 4).

## DISCUSSION

Psychosocial factors are able to exert influence on deterioration of disease, symptoms, responses to treatment, and the quality of life. Researchers evaluated the psychological status of patients with chronic disease using SCL-90-R questionnaire. A link of GERD and psycho-pathological features has already been demonstrated by several studies.^14,15^ We analyzed the data from the patients with refractory GERD symptoms in order to acquire their psychological characteristics, which may be helpful for making diagnosis and clinical strategy.

In this study, O-S and I-S, which are stable personality characters and are referred as personality, were not found to be statistically significant between these patients. But, differences were found regarding DEP, ANX, PAR, PSY, and GSI domains. The results suggest that the psychological status perhaps does not have close relationship with acid reflux. Remarkably, achalasia showed more psychological factors. Considering that achalasia is an unknown congenital disease, those factors will certainly exist because of refractory GERD symptoms. They probably are the result of symptoms from which patients have suffered for a quite long duration. Taken together, it appears that psychological factors will have a distinct effect on chronic diseases.^5,16,17^

With our other comparison results based on DeMeester value, we did not find difference between pathological and normal acid reflux. This strongly supports our suggestion that psychological status does not relate to acid reflux again. Possible reason is that the universal usage of PPIs has effectively controlled acid reflux; therefore, this makes patients benefit from reducing mental stress.

A large number of previous studies have revealed that patients with typical symptoms are apt to suffer from atypical GERD symptoms and FGID symptoms.^18,19^ Also, there is always an overlap between reflux symptoms, irritable bowel syndrome, and functional dyspepsia. The atypical GERD symptoms are closely related to the typical GERD symptoms.^20^ Unlike the study of Lee et al^21^ that showed that patients with symptomatic RE had more scores than patients with asymptomatic RE on all dimensions of SCL-90-R, we chose patients only with typical GERD symptom to compare with those with typical plus atypical GERD symptom. Our aim was to acquire more precise information about the relationship between mental disorder and GERD symptom.

The analysis of symptom duration in this study showed that the patients with more duration had more problems regarding DEP, ANX, and PSY. Considering gender difference, we investigated the data divided by females and males. Our results are consistent with those of Núñez-Rodríguez and Miranda Sivelo^17^ that women had dramatically higher scores than men in these nine domains by SCL-90-R. Due to more and more researchers paying attention to gender difference in chronic disease development,^22-24^ we assume that the difference between females and males is worthy of further investigations.

Regarding the measurement of mental disorders with SCL-90-R, some limitations are also prevailing in this study. The processes including interviewing, diagnosing, and rating were done by experts in our gastroenterology department. Although our research team acquired the assistance of psychologists, the results were likely to have a little bias. Moreover, the samples in this study were regional instead of a being nationally representative. Differences across cities and countries may occur due to differences in sociodemographics. Large-scale multicenter studies need to be carried out in future.
